# Silibinin Alleviates Liver Oxidative Stress in D-Galactose-Treated Kunming Mice via Microbiota Homeostasis in a Microbiota-Dependent Manner

**DOI:** 10.3390/antiox14091087

**Published:** 2025-09-05

**Authors:** Ao Dong, Xianglong Zhang, Jianxiang Ma, Jiahui Cao, Gnagna Diene, Yiou Xu, Xiujie Yin, Yang Li, Yonggen Zhang

**Affiliations:** College of Animal Science and Technology, Northeast Agricultural University, Harbin 150030, China; 18745769350@163.com (A.D.); zxl16695190908@163.com (X.Z.); 15776845817@163.com (J.M.); 17612845180@163.com (J.C.); gnagnadiene01@gmail.com (G.D.); xvyiou@163.com (Y.X.); yinxiujie@126.com (X.Y.)

**Keywords:** silibinin, oxidative stress, D-galactose, cecal microbiota, metabolome

## Abstract

Hepatic oxidative stress is a key driver in liver injury pathogenesis, with D-galactose (D-gal) modeling serving as an established inducer of accelerated oxidative damage. Silibinin (SLB), a flavonolignan from milk thistle, shows therapeutic promise through potent antioxidant activity and gut–liver axis modulation. This study investigated whether the hepatoprotective effect of SLB against oxidative stress depends on gut microbiota regulation. Using mouse models with gut microbiota ablation by oral antibiotics or direct oxidative stress induction by D-gal (150 mg/kg), SLB treatment (200 mg/kg) was administered. The protective mechanisms were evaluated through the Nrf2/ARE pathway, target gene expression, gut microbiota profiling, and cecal metabolomics. Results demonstrated that SLB significantly alleviated D-gal-induced hepatic oxidative stress (e.g., reduced MDA by 33.3%), but this protection was markedly weakened after antibiotic-induced microbiota depletion (e.g., a loss of efficacy exceeding 50%). Integrated omics revealed that antibiotics caused a severe reduction in unclassified_Muribaculaceae (a butyrate producer, decreased by 80%), impairing butyrate-mediated Nrf2/Keap1 activation. Simultaneously, the absence of Parabacteroides led to accumulated primary bile acids and inhibited secondary bile acid production (e.g., taurochenodeoxycholate reduced by 75%), further disrupting redox homeostasis. Conclusion: Silibinin’s mitigation of hepatic oxidative stress is gut microbiota-dependent, highlighting the therapeutic potential of microbiota-targeted antioxidant strategies for oxidative stress-related pathologies.

## 1. Introduction

Oxidative stress occurs when the body generates an excess of reactive oxygen species (ROS) that surpasses the ability of its antioxidant defenses to neutralize them. These ROS can harm cellular components, including lipids, proteins, and DNA, leading to cellular damage, inflammation, and genetic mutations [1,2]. The harmful effects of oxidative stress can accelerate tissue aging, impair normal function, and contribute to the development of various diseases, including cardiovascular disorders, diabetes, cancer, and Alzheimer’s disease [3,4,5]. As a result, oxidative stress is thought to be a major cause of aging and a number of chronic illnesses. Therefore, effective prevention and treatment of oxidative stress are essential for maintaining health and slowing down aging.

Emerging evidence highlights the gut microbiota as a pivotal regulator in both intestinal and systemic pathophysiological processes. The liver, as the organ most intimately connected with the gut via the enterohepatic circulation through anatomical connections including the biliary tract, portal vein, and systemic circulation, is persistently exposed to gut-derived bacterial components and metabolites [6]. This unique anatomical relationship explains the frequent association between gut microbiome alterations and hepatic pathologies. Hepatocytes, despite their robust antioxidant defenses, exhibit heightened susceptibility to ROS-mediated oxidative damage due to their central role in detoxification and metabolic regulation. Chronic hepatic oxidative stress thus constitutes a key etiological factor in liver diseases [7,8]. Furthermore, gut microbiota integrity governs nutrient absorption, immune development, and barrier function. Age-related oxidative stress disrupts microbial homeostasis, compromises intestinal barrier integrity, and facilitates portal translocation of inflammatory mediators, exacerbating hepatic oxidative injury [9,10]. These observations position gut–liver axis homeostasis as a promising therapeutic target for mitigating liver oxidative stress.

Probiotics, medicinal plants and their extracts have shown good efficacy in alleviating damage caused by oxidative stress [11,12]. SLB (C_25_H_22_O_10_) is the principal active compound extracted from the fruit of Silybum marianum, a plant of the Asteraceae family, and belongs to the flavonoid lignans [13]. As a natural plant compound, SLB possesses immunomodulatory, antioxidant and anti-inflammatory bioactivities. Extensive evidence from preclinical studies (including animal and cellular research), as summarized in reviews, supports its role in protecting the liver and improving liver function, suggesting its potential efficacy in the treatment of alcohol-, drug- or virus-induced liver damage [14,15,16]. The mechanisms underlying these effects, particularly evidenced by cellular studies [16], include direct scavenging of free radicals, enhancement of endogenous antioxidant defense system, inhibition of lipid peroxidation to maintain cellular membrane integrity, stabilization of mitochondrial function, and reduction in ROS production [17,18]. Although SLB is known for its direct antioxidant effects, its role in modulating gut flora to alleviate hepatic oxidative stress remains poorly characterized.

Based on this, we hypothesized that SLB exerts its hepatoprotective effects in a gut microbiota-dependent manner. To test this hypothesis, our study was designed to compare the efficacy of SLB in mice with an intact microbiome versus those subjected to antibiotic-induced microbiota depletion. The role of the gut microbiota was evaluated by assessing key antioxidant pathways alongside comprehensive microbial and metabolomic profiling [19]. This study investigates whether SLB can alleviate D-gal-induced hepatic oxidative stress in a microbiota-dependent manner.

## 2. Materials and Methods

### 2.1. Drugs and Reagents

Silibinin was sourced from Huacheng Pharmaceutical Co., Ltd. (Shenyangng, China). D-Galactose and antibiotics (ampicillin, neomycin sulfate, streptomycin sulfate) were acquired from Macklin (Shanghai, China). Biochemical assay kits for total superoxide dismutase (T-SOD), total antioxidant capacity (T-AOC), catalase (CAT), malondialdehyde (MDA), and glutathione peroxidase (GSH-Px) were supplied by NJJCBIO (Nanjing, China). Chromatographic-grade solvents including formic acid, methanol, and acetonitrile were procured from Thermo Fisher Scientific (Waltham, MA, USA). The internal standard L-2-Chlorophenylalanine was obtained from HCBioTech (Shanghai, China). All chemicals and solvents met analytical or HPLC-grade specifications.

### 2.2. Animals and Drug Administration

**Figure 1:**
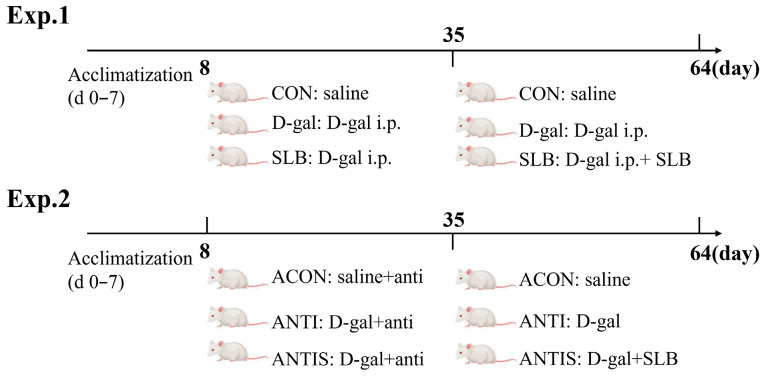
Experimental design. Schematic of the experimental timeline showing acclimation (d 0–7), antibiotic treatment period (d 8–35, Exp. 2 only), and D-galactose induction (d 8–64) with or without SLB supplementation.

Animal Experimental Design: Sixty female Kunming mice (6-week-old; Changsheng Biotechnology, China) were acclimatized for 7 days in specific pathogen-free (SPF) conditions (20–24 °C, 45–60% humidity, 12 h light/dark cycle) within individually ventilated cages (IVCs; 4 mice/cage). All animal procedures were approved by the Northeast Agricultural University Animal Care and Use Committee (NEAUEC20240252) and were conducted in accordance with the National Research Council’s (NRC) guidelines for the care and use of laboratory animals. Mice were randomly allocated to two parallel experiments, each comprising 30 mice with 10 mice per group: Experiment 1 (Aging Model) consisted of the CON group (control, saline oral gavage; *n* = 10), D-gal model group (150 mg/kg/day D-gal i.p.; *n* = 10), and SLB group (D-gal + 200 mg/kg/day SLB oral gavage; *n* = 10); Experiment 2 (Pseudosterile Model) included the ACON group (antibiotics + saline gavage; *n* = 10), ANTI group (antibiotics + D-gal; *n* = 10), and ANTIS group (antibiotics + D-gal + SLB; *n* = 10). Days 0–7 served as an acclimation period without interventions. During days 8–35: Experiment 1: CON received saline oral gavage, while D-gal and SLB groups received daily D-gal i.p. (150 mg/kg). Experiment 2: All groups consumed antibiotic water (1 g/L ampicillin-neomycin-streptomycin, refreshed every 48 h). ACON received saline oral gavage, while ANTI and ANTIS received D-gal i.p. During days 36–64: Experiment 1: CON continued saline oral gavage; D-gal group continued D-gal i.p.; SLB group received D-gal i.p. plus SLB oral gavage. Experiment 2: Antibiotic water was discontinued; ACON continued saline oral gavage; ANTI continued D-gal i.p.; ANTIS received D-gal i.p. plus SLB oral gavage. The experimental timeline is summarized schematically in Figure 1.

### 2.3. Body Weight and Organ Index

Weekly body mass measurements were recorded for all experimental subjects. Following final weight measurements 24 h post-treatment, animals were euthanized and subjected to necropsy procedures. Harvested organs underwent saline perfusion (0.9% NaCl solution, *w*/*v* ratio 1:5) to remove blood residues. Spleen, thymus, and hepatic tissues were precisely weighed to compute relative organ indices, expressed as tissue mass (milligrams) per kilogram of total body mass [20].

### 2.4. Hematoxylin and Eosin Staining

Hematoxylin–eosin (H&E) staining procedures followed established protocols. Rat liver tissues underwent fixation in 4% paraformaldehyde–PBS solution for 24 h before sequential processing through dehydration and paraffin embedding [21]. Following dehydration and paraffin embedding, tissue sections of 5 μm thickness were prepared using a Leica RM2235 rotary microtome, processed through deparaffinization and rehydration steps, and examined under a Nikon Eclipse E100 LED microscope at 400× magnification before undergoing H&E staining.

### 2.5. Antioxidant Capacity Detection

The concentrations and enzymatic activities of T-SOD, T-AOC, GSH-Px, MDA, and CAT in hepatic tissues from all 10 mice per group were assessed alongside serum levels of T-SOD, T-AOC, GSH-Px, and MDA using the same biological replicates (*n* = 10) following commercial kit protocols.

### 2.6. Western Blotting Analysis

Protein samples from three biological replicates per group were mixed with radioimmunoprecipitation assay (RIPA) buffer supplemented with a protease inhibitor cocktail and thoroughly homogenized. Following homogenization, the samples underwent centrifugation, after which the resulting supernatants were carefully collected. Protein quantification was performed using the bicinchoninic acid assay (BCA) method. Protein separation was achieved through sodium dodecyl sulfate–polyacrylamide gel electrophoresis (SDS-PAGE), followed by electrophoretic transfer onto nitrocellulose membranes. Subsequently, membranes were probed with primary antibodies targeting glutathione peroxidase 4 (GPX4, 1:2000), nuclear heme oxygenase-1(HO-1, 1:1000), Keap1(1:2000), Nrf2(1:1000), NADH dehydrogenase quinone 1(NQO1, 1:1000), and glyceraldehyde-3-phosphate dehydrogenase (GAPDH, 1:5000, loading control), with incubation carried out overnight at 4 °C. After incubation with horseradish peroxidase-conjugated secondary antibodies (anti-rabbit, 1:5000; anti-mouse, 1:5000), proteins were detected by enhanced chemiluminescence (ECL) with 1 min film exposures in a darkroom, and signals from three technical replicates per blot were quantified using ImageJ (version 1.53m) software.

### 2.7. Quantitative RT-PCR

The mRNA levels of GPX4, HO-1, Keap1, Nrf2, and NQO1 were quantified through quantitative real-time polymerase chain reaction. Hepatic RNA was isolated using the TRIzol reagent and subsequently synthesized into complementary DNA through reverse transcription. Amplification reactions employed primers listed in Table S1, following the supplier’s guidelines (Sagon Biotech, Shanghai, China). Thermal cycling parameters consisted of initial denaturation at 95 °C for 30 s, followed by 40 cycles of denaturation at 95 °C for 5 s and annealing/extension at 60 °C for 34 s. A final extension step was performed at 72 °C for 3 min.

Gene expression levels were measured using β-actin served as the housekeeping gene.

### 2.8. Colonic Content DNA Extraction and 16 S rRNA Gene Sequencing

Murine fecal genomic DNA extraction was performed according to manufacturer specifications using the TGuide S96 Magnetic Soil/Stool DNA Kit (Tiangen Biotech, Beijing, China). DNA integrity assessment employed 1.8% agarose gel electrophoresis, while quantification of concentration and purity (A_260_/_280_ and A_260_/_230_ ratios) utilized a NanoDrop 2000 spectrophotometer (Thermo Fisher Scientific, Wilmington, DE, USA). Amplification of bacterial 16S rRNA gene V3-V4 hypervariable regions was conducted with indexed primers 338F (5′-ACTCCTACGGGAGGCAGCA-3′) and 806R (5′-GGACTACHVGGGTWTCTAAT-3′) in 10 μL reaction volumes containing: 5–50 ng DNA template, 0.3 μL each of 10 μM forward and reverse primers, 5 μL KOD FX Neo Buffer, 2 μL of 2 mM dNTP mixture, and 0.2 μL KOD FX Neo polymerase. Thermal cycling parameters consisted of initial denaturation at 95 °C for 5 min, followed by 20 cycles of denaturation (95 °C, 30 s), annealing (50 °C, 30 s), and extension (72 °C, 40 s), with a final extension at 72 °C for 7 min. Purification of amplicons was achieved with the Omega DNA system (Omega Bio-Tek, Norcross, GA, USA; Cat. No. D01-100.), subsequent quantification performed on a Qsep-400 instrument (BiOptic Inc., New Taipei City, Taiwan, China; Software v2.6.1), and paired-end sequencing (250 bp reads) executed on an Illumina NovaSeq 6000 platform (Illumina Inc., San Diego, CA, USA; Beijing Biomarker Technologies). Bioinformatics processing via the BMKCloud platform (https://www.biocloud.net, accessed on 20 June 2024) encompassed: (1) Raw data quality control using FastQC v0.11.9. (2) Chimera removal with UCHIME algorithm (USEARCH v10.0). (3) Operational taxonomic unit (OTU) clustering at 97% similarity through USEARCH v10.0. (4) Rarefaction to 30,000 sequences per sample for alpha/beta diversity normalization. (5) Taxonomic assignment implementing QIIME2′s Naive Bayes classifier against SILVA v138.1 (70% confidence). (6) Alpha diversity indices (e.g., Shannon, Simpson) were calculated. (7) Beta diversity visualization via principal coordinate analysis (PCoA) [22]. (8) Statistical comparison using one-way ANOVA.

### 2.9. Colonic Content Metabolomics

Fecal metabolites were extracted from 50 mg aliquots in pre-chilled 1.5 mL tubes containing stainless steel beads. Samples were homogenized with 1000 μL of ice-cold extraction solvent (methanol/acetonitrile/water = 2:2:1, *v*/*v*/*v*) containing 2-chloro-L-phenylalanine internal standard (2 mg/L). Homogenization was performed by mechanical milling (45 Hz, 10 min), followed by ice-bath sonication (10 min) and incubation (−20 °C, 1 h). After centrifugation (12,000 rpm, 4 °C, 15 min), a 500 μL aliquot of supernatant was lyophilized. The dried residue was reconstituted sequentially in 800 μL acetonitrile and 800 μL water, followed by vortex mixing (30 s), ice-bath sonication (10 min), and recentrifugation under identical conditions.

Processed supernatants (120 μL) were transferred for UPLC-MS analysis. Quality control (QC) samples were prepared by pooling 10 μL aliquots from each sample extract. Chromatographic separation was achieved using a Waters UPLC system equipped with an ACQUITY UPLC HSS T3 column (100 × 2.1 mm, 1.8 μm; maintained at 45 °C) and guard column. The mobile phase consisted of (A) 0.1% aqueous formic acid and (B) 0.1% formic acid in acetonitrile, delivered at 0.4 mL/min with the following gradient: 2% B (0.25 min), linear increase to 98% B (9.75 min), hold at 98% B (3 min), return to 2% B (0.1 min), and equilibration at 2% B (1.9 min; total run time: 15 min). Injection volume was 1 μL.

Mass spectrometric detection was performed using a QE high-resolution mass spectrometer operating in both positive and negative ESI modes. Key parameters included: *m/z* range 100–1200, resolution 70,000 (MS) and 17,500 (MS/MS), stepped collision energies (10, 20, 40 eV), spray voltages (2.5 kV ESI^+^, 2.0 kV ESI^−^), sheath gas (40 AU), auxiliary gas (10 AU), and capillary temperature (320 °C).

Raw data (acquired using MassLynx V4.2) were processed in Progenesis QI for peak detection, alignment, and feature extraction. Data were normalized to internal standards and total ion current to correct for instrument variability. Metabolite identification integrated METLIN database matching, interrogation of the BMKCloud repository, and theoretical fragmentation pattern verification (mass tolerance < 100 ppm).

### 2.10. Statistical Analysis

Statistical analyses were performed using GraphPad Prism 6 software, with quantitative data expressed as mean ± SD. Normality (Shapiro–Wilk test) and homogeneity of variance (Levene’s test) were confirmed for all datasets prior to parametric testing. For experimental groups containing three or more samples, inter-group comparisons were conducted through one-way ANOVA, followed by Tukey’s post hoc test for multiple comparisons. Differences between two experimental conditions were evaluated using Student’s *t*-test. The predetermined threshold for statistical significance was set at *p* < 0.05.

## 3. Results

### 3.1. Effects of Silibinin on Body Weight and Organ Index in Mice with D-Galactose Oxidative Damage

**Figure 2:**
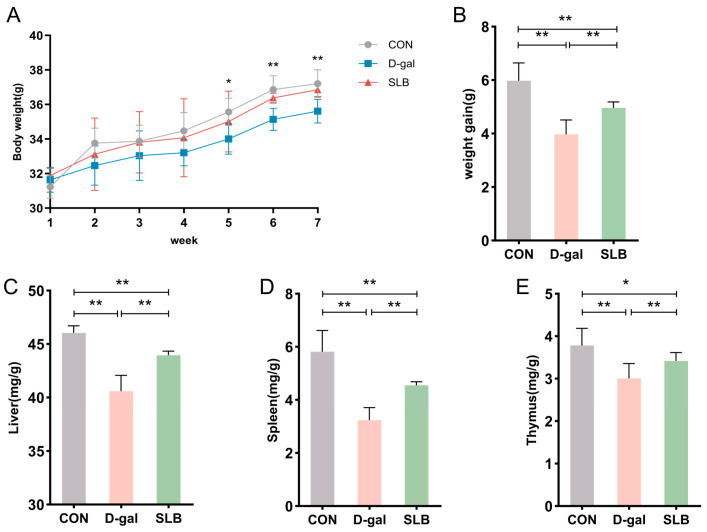
Silibinin counteracts D-gal effects on body weight and organ indices. (**A**) Changes in body weight of mice during the course of the experiment. (**B**) Net change in body weight of mice. (**C**) Liver index of mice at day 57. (**D**) Spleen index of mice at day 57. (**E**) Thymus index of mice at day 57. * *p* < 0.05, ** *p* < 0.01.

During the 7-week observation period, mice in the normal control group maintained active behavior, exhibited glossy fur, demonstrated normal food intake, and showed progressive weight gain. In contrast, compared to the control group, the D-gal-treated group displayed delayed responsiveness, unkempt fur, decreased appetite, and diminished weight gain compared to controls. Compared to the D-gal group, silibinin intervention (SLB group) produced noticeable improvements in phenotypic characteristics from week 5 onward (Figure 2A), accompanied by significantly enhanced weight gain relative to the D-gal group (Figure 2B).

Organ weight analysis revealed significantly reduced liver indices in the model group relative to controls (Figure 2C), which was partially reversed by SLB treatment. Similar patterns were observed in spleen and thymus indices, with the model group showing decreased values (Figure 2D,E) that were notably elevated in the SLB group.

Collectively, compared to the model group, the SLB-treated group demonstrated marked increases in liver, spleen, and thymus indices compared to the model group, indicating the compound’s potential to counteract D-gal-induced organ aging.

### 3.2. Effects of Silibinin on Oxidative Stress in the Liver and Blood Induced by D-Galactose in Mice

**Figure 3:**
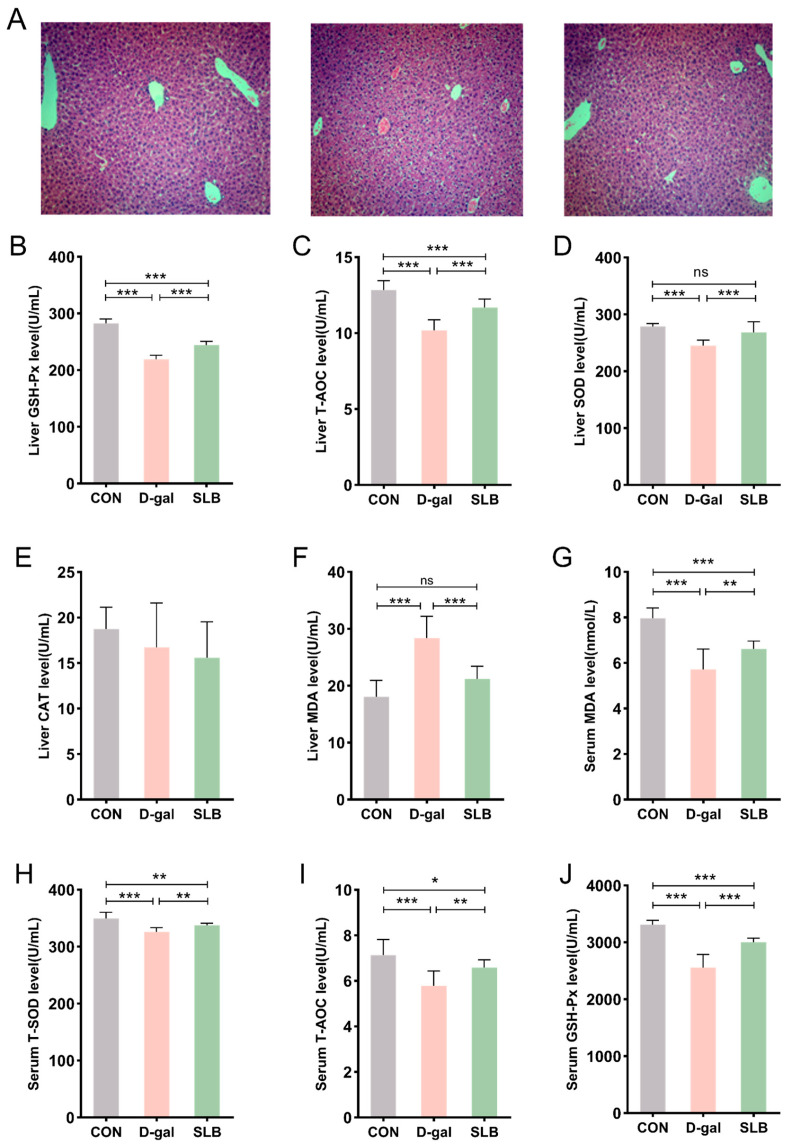
Therapeutic efficacy of silibinin on D-gal-induced multilevel oxidative injury. (**A**) H&E staining of oxidative stress injury in liver tissue at day 57. (**B**–**F**) GSH-Px, T-AOC, SOD, CAT, and MDA levels in mouse liver. (**G**–**J**) The levels of MDA, T-SOD, T-AOC, and GSH-Px in the serum of mice were measured. Histological Activity Index (HAI) scores for each group. * *p* < 0.05, ** *p* < 0.01, *** *p* < 0.001, ns, not significant.

H&E staining of liver tissues across experimental groups is presented in Figure 3A. Control (CON) group specimens exhibited intact hepatic architecture with clearly visible nuclei. In contrast, D-gal-treated mice demonstrated structural abnormalities including hepatocyte swelling, cytoplasmic vacuolation, and nuclear irregularities when compared to the CON group. These pathological alterations induced by D-gal were significantly ameliorated in the SLB-treated (SLB) group compared to the D-gal group. This improvement was supported by a significant reduction in Histological Activity Index (HAI) scores in the SLB group compared to the D-gal model group (Figure 3K).

Hepatic oxidative stress assessment revealed that compared to the CON group, the D-gal group had significantly reduced activities of GSH-Px, T-AOC, and SOD, accompanied by elevated MDA levels (Figure 3B–F). Importantly, compared to the D-gal group, SLB administration effectively reversed these oxidative stress markers, though no significant differences in CAT activity were observed between any of the experimental groups.

Systemic evaluation of oxidative stress biomarkers in serum displayed analogous patterns (Figure 3G–J). Compared to the CON group, the D-gal group showed decreased serum antioxidant enzyme activities (GSH-Px, T-AOC, SOD) and increased MDA content relative to controls. These D-gal-induced alterations in systemic oxidative stress parameters were significantly normalized following SLB intervention compared to the D-gal group.

### 3.3. Silibinin Attenuates D-Galactose-Induced Liver Injury and Modulates Associated Blood Biochemistry and Immune Responses

**Figure 4:**
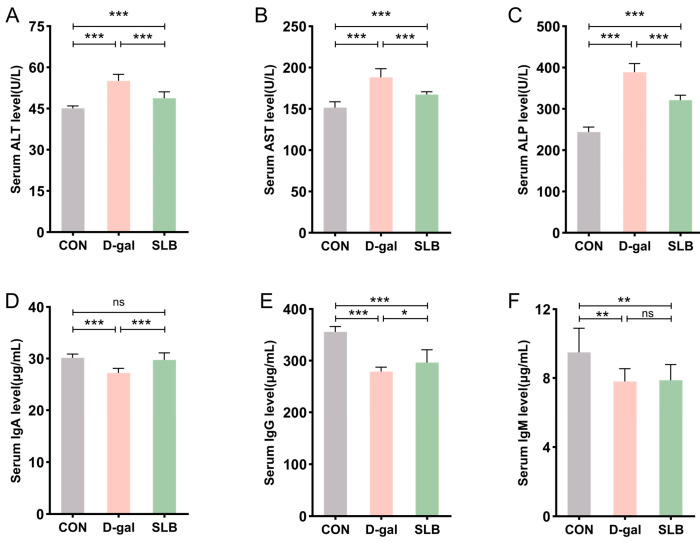
Silibinin normalizes D-gal-perturbed serum hepatotoxic and immunogenic parameters. (**A**–**C**) The levels of ALT, AST and ALP in mice serum. (**D**–**F**) The levels of IgA, IgG and IgM in mice serum. * *p* < 0.05, ** *p* < 0.01, *** *p* < 0.001, ns, not significant.

Serum biomarkers of hepatic injury (ALT, AST, ALP) showed significant elevation in the D-gal group compared to controls (CON), consistent with impaired liver function. Compared to the D-gal group, SLB administration substantially attenuated these biochemical alterations (Figure 4A–C).

Assessment of humoral immunity through immunoglobulin quantification revealed marked reductions in serum IgA, IgG, and IgM levels in the D-gal group relative to controls. Compared to the D-gal group, while SLB treatment significantly restored IgA and IgG concentrations, IgM levels demonstrated only marginal improvement (Figure 4D–F).

### 3.4. Silibinin Modulates Expression of Nrf2/Keap1 Pathway Proteins and mRNAs

The Nrf2/Keap1 signaling pathway critically regulates cellular antioxidant responses through ARE activation. To evaluate its role in D-gal-induced oxidative stress, hepatic expression of Nrf2/Keap1 pathway components was assessed at both protein and mRNA levels.

**Figure 5:**
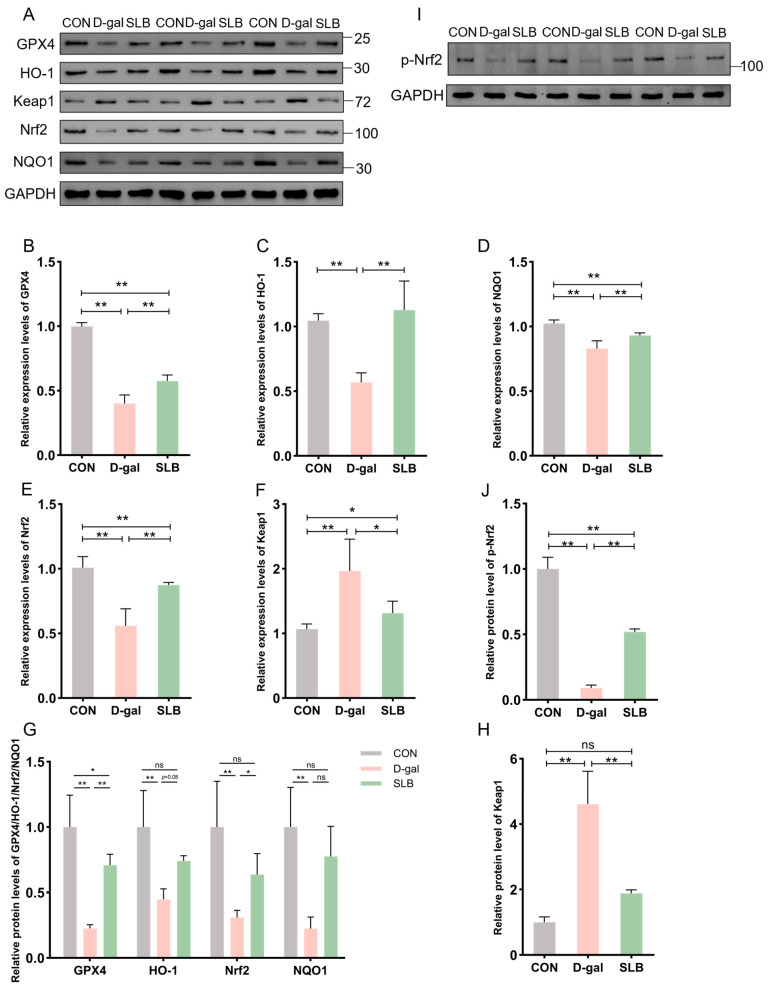
Pharmacological reactivation of compromised Nrf2 antioxidant system by silibinin. (**A**) Western blot for GPX4, HO-1, Keap1, Nrf2, and NQO1 of murine liver tissue. (**B**–**F**) The mRNA abundance of Nrf2 signaling components (GPX4, HO-1, Keap1, NQO1) and Nrf2. (**G**,**H**,**J**) GPX4, HO-1, Nrf2, NQO1, Keap1 and p-Nrf2 protein expression was quantified. (**I**) Western blot for p-Nrf2 of murine liver tissue. * *p* < 0.05, ** *p* < 0.01, ns, not significant. GPX4 (glutathione peroxidase 4), HO-1 (nuclear heme oxygenase-1), NQO1 (NADH dehydrogenase quinone 1).

Relative to controls, D-gal administration significantly suppressed Nrf2 protein expression while elevating Keap1 levels compared to controls (CON). Consistently, the phosphorylation level of Nrf2 was also decreased by D-gal exposure. This suppression was effectively reversed by SLB treatment effectively restored nuclear Nrf2 translocation and p-Nrf2 expression, and reduced Keap1 accumulation (Figure 5A,G–J). Downstream targets GPX4, HO-1, and NQO1 exhibited parallel protein suppression in the D-gal group compared to CON levels. Following SLB intervention, GPX4 expression was significantly upregulated, while HO-1 and NQO1 showed only partial restoration relative to the D-gal model (Figure 5A,G).

At the mRNA level, D-gal treatment decreased expression of GPX4, HO-1, NQO1, and Nrf2 while increasing Keap1 expression compared to control levels. These transcriptional alterations were largely normalized by SLB intervention (Figure 5B–F).

### 3.5. Effects of Silibinin on Gut Microbiota Composition and Untargeted Metabolites in Mice with D-Galactose Oxidative Damage

**Figure 6:**
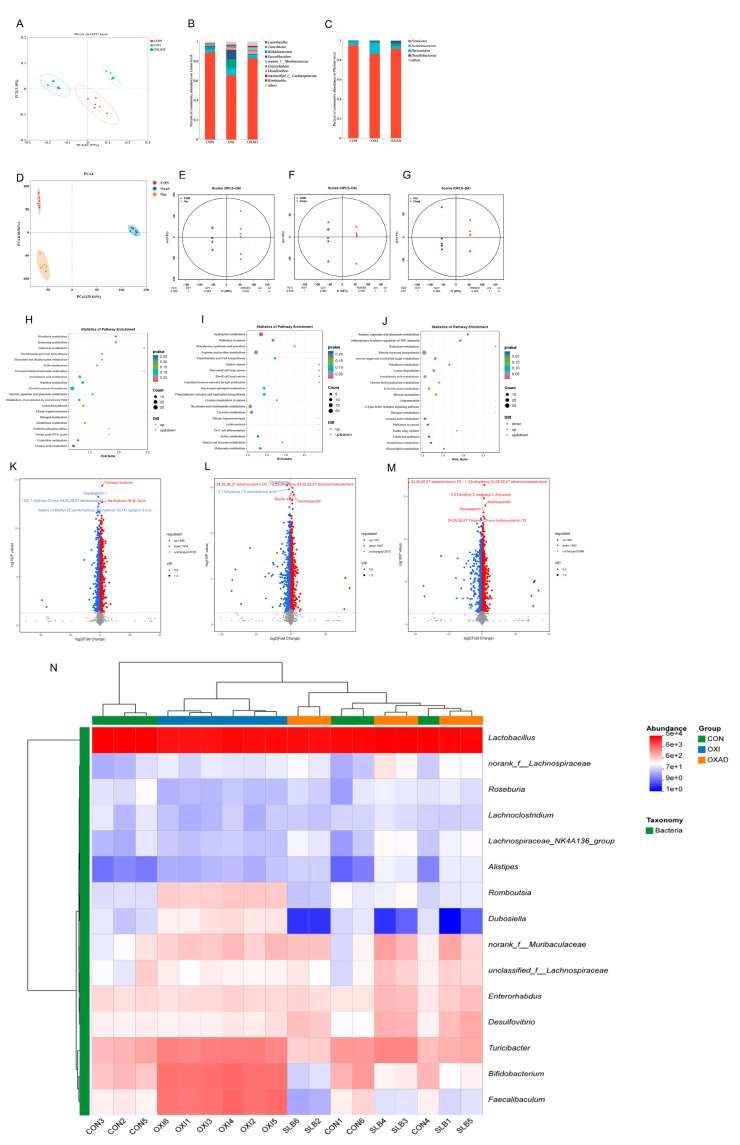
Integrated analysis of cecal microbiota and metabolites across control (CON), D-galactose-induced (D-gal), and Silibinin-treated (SLB) groups. (**A**) Microbial β-diversity assessed by Principal Coordinates Analysis (PCoA). (**B**) Phylum-level relative abundance distribution. (**C**) Genus-level relative abundance distribution. (**D**) Metabolomic β-diversity (PCoA) of pairwise group comparisons. (**E**–**G**) Orthogonal Projections to Latent Structures-Discriminant Analysis (OPLS-DA) score plots. (**H**–**J**) KEGG pathway enrichment of differential metabolites: CON vs. D-gal (**H**), CON vs. SLB (**I**), D-gal vs. SLB (**J**). (**K**–**M**) Volcano plots of differential metabolites: CON vs. D-gal (**K**), CON vs. SLB (**L**), D-gal vs. SLB (**M**). (**N**) Heatmap of the relative abundance of gut microbiota at the genus level.

Analysis of cecal bacterial alpha diversity (Table 1) revealed that SLB administration significantly increased (*p* < 0.05) the Shannon index, abundance-based coverage estimator (ACE), and Chao1 index compared to the D-galactose (D-gal) oxidative stress model group. Assessment of beta diversity demonstrated significantly greater inter-group variation than intra-group variation (Figure 6A). Principal coordinates analysis (PCoA) clearly segregated the three groups (CON, D-gal, SLB), with the control (CON) and SLB groups clustering more closely to each other than to the D-gal group, indicating a partial restoration of gut microbiota composition by SLB treatment. Relative to the D-gal group, *Firmicutes*, *Bacterodota*, and *Desulfobacterota* were substantially more abundant in the SLB group than in the D-gal group at the phylum level, but *Actinobacteriota* was significantly less abundant (Figure 6B, Table 2, *p* < 0.05). Genus-level analysis further showed that SLB treatment significantly increased the abundances of *Lactobacillus*, *norank_Muribaculaceae*, *Desulfovibrio*, *Enterorhabdus*, and *unclassified_Lachnospiraceae*, while significantly decreasing *Bifidobacterium*, *Turicibacter*, *Faecalibaculum*, and *Romboutsia* compared to the D-gal model (Figure 6C, Table 2, *p* < 0.05).

Untargeted metabolomics detected 7460 metabolites in cecal contents. Pairwise comparisons identified significant differences: 3332 differential metabolites (1488 up/1844 down) between CON and D-gal groups, 3548 (1741 up/1807 down) between CON and SLB groups, and 3472 (1867 up/1605 down) between D-gal and SLB groups. Multivariate analysis demonstrated distinct metabolite profiles across groups via PCoA (Figure 6D). OPLS-DA models confirmed significant separations in all comparisons (Figure 6E–G), with high model validity (R^2^Y = 1, Q^2^Y > 0.984). Volcano plots visualized differential metabolite distributions (Figure 6K–M). KEGG enrichment analysis (*p* < 0.05) identified oxidative stress-associated pathways including Butanoate, Riboflavin, Galactose, and Tryptophan metabolism; Aldosterone synthesis/secretion; Alanine, aspartate and glutamate metabolism; Inflammatory mediator regulation of TRP channels; and Pathways in cancer (Figure 6H–J, Table 3). Critically, compared to the D-gal group, SLB intervention ameliorated D-gal-induced metabolic dysregulation, exemplified by significant changes in 2-Oxoglutaramate and L-Glutamine (Alanine/aspartate/glutamate metabolism), 15(S)-HPETE and Icilin (TRP channel regulation), and 3-beta-D-Galactosyl-sn-glycerol (Galactose metabolism) (Tables S1–S3).

### 3.6. Effects of Silibinin on Body Weight and Organ Index in Mice with D-Galactose Oxidative Damage After Antibiotic Clearance

**Figure 7:**
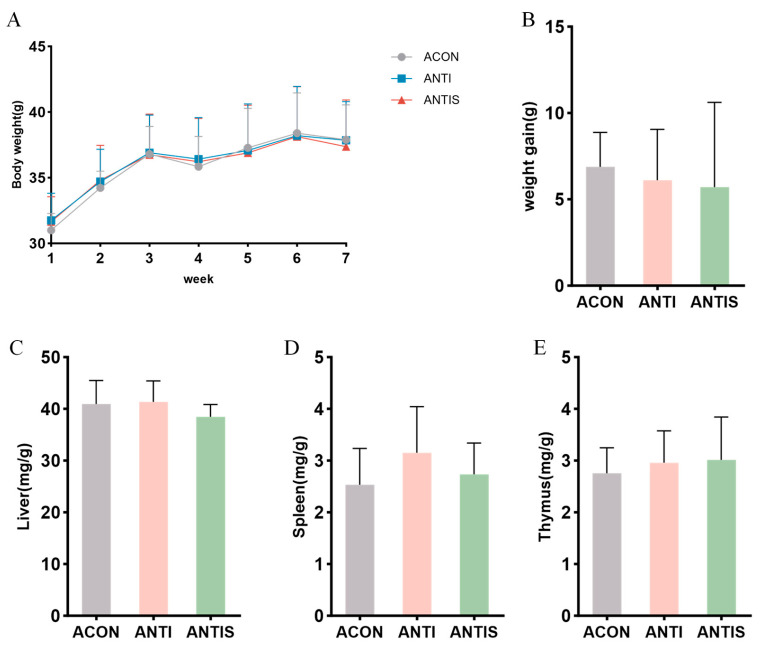
Gut microbiota depletion blunts the mitigatory effects of silibinin on D-galactose-induced physiological decline. (**A**) Changes in body weight of mice during the course of the experiment. (**B**) Net change in body weight of mice. (**C**) Liver index of mice at day 57. (**D**) Spleen index of mice at day 57. (**E**) Thymus index of mice at day 57.

After antibiotic treatment, the weight change curve and weight gain of the mice did not change significantly throughout the experimental period (Figure 7A,B). Moreover, the weight changes in liver, spleen and thymus were not significantly different among the three groups (Figure 7C–E).

### 3.7. Effects of Silibinin on Oxidative Stress Indices in the Liver and Serum of Mice with D-Galactose-Induced Damage After Antibiotic Clearance

**Figure 8:**
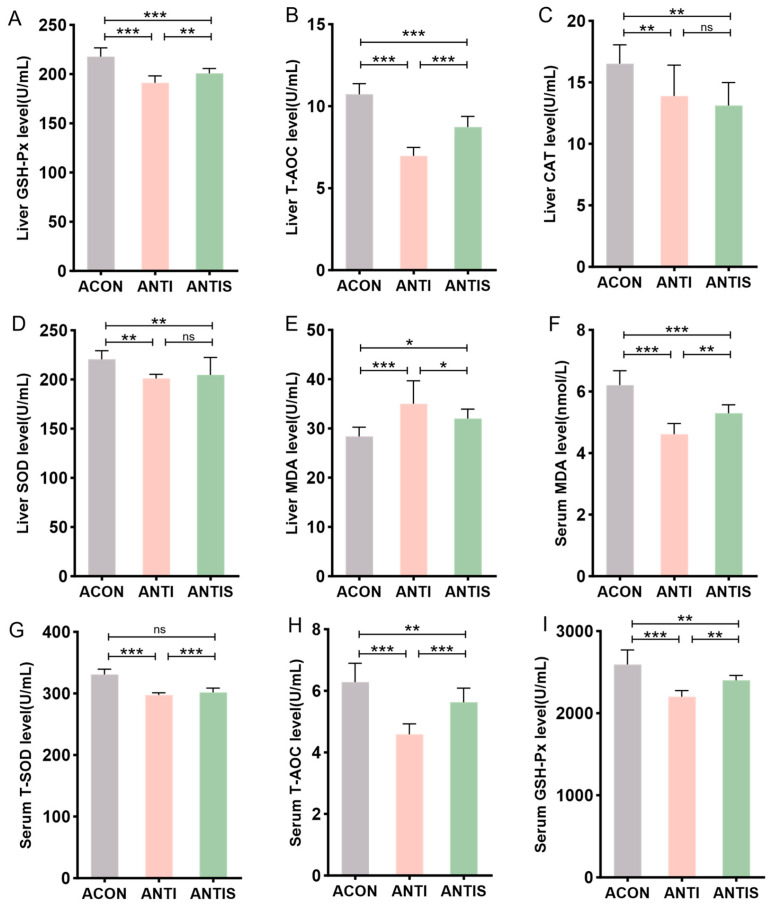
Assessment of silibinin effects on D-galactose-induced multilevel oxidative injury under antibiotic-mediated gut microbiota depletion. (**A**–**E**) GSH-Px, T-AOC, CAT, SOD, and MDA levels in mouse liver. (**F**–**I**) The levels of MDA, T-SOD, T-AOC, and GSH-Px in the serum of mice were measured. * *p* < 0.05, ** *p* < 0.01, *** *p* < 0.001, ns, not significant.

To investigate intestinal microbiota involvement in SLB-mediated hepatic antioxidant effects, antibiotic-treated (ANTI) mice received SLB supplementation. Hepatic analysis demonstrated significantly reduced activities of GSH-Px, T-AOC, CAT, and SOD in the ANTI group relative to controls (ACON), with concurrent elevation of MDA levels. Compared to the ANTI group, SLB treatment normalized hepatic GSH-Px, T-AOC, and MDA parameters (Figure 8A,B,E), whereas CAT and SOD activities remained unaltered (Figure 8C,D).

Systemic oxidative stress assessment revealed analogous patterns: the antibiotic + stress (ANTIS) group exhibited elevated serum MDA (+30%) and diminished T-SOD (−20%), T-AOC (−45%), and GSH-Px activities compared to antibiotic controls (ACON). Relative to the ANTIS group, SLB intervention partially restored these serum biomarkers; for instance, serum GSH-Px activity was improved by approximately 15% (Figure 8F–I).

Collectively, these findings implicate intestinal microbiota in facilitating SLB’s mitigation of hepatic oxidative stress.

### 3.8. Effects of Silibinin on Liver Injury Markers in Blood Biochemistry and Immune Indices of Mice with D-Galactose-Induced Damage After Antibiotic Clearance

**Figure 9:**
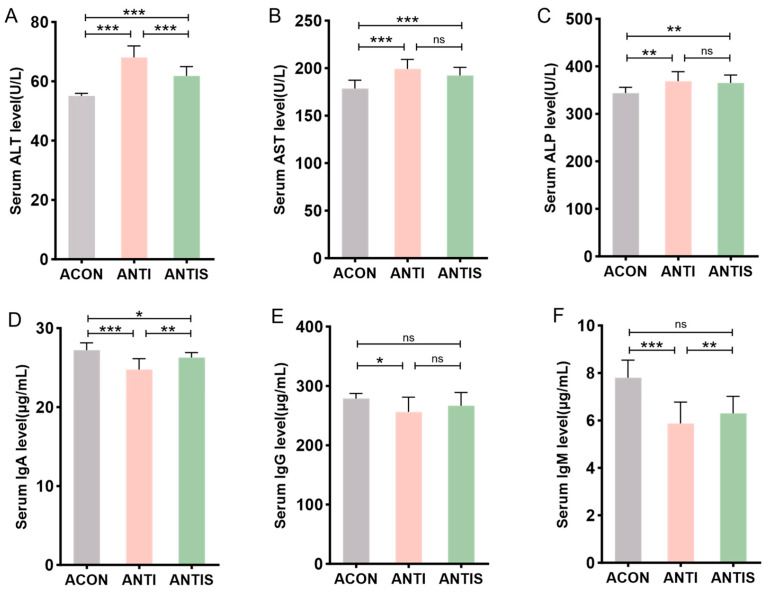
Serum biomarkers in microbiota-depleted mice with D-galactose and silibinin interventions. (**A**–**C**) The levels of ALT, AST and ALP in mice serum. (**D**–**F**) The levels of IgA, IgG and IgM in mice serum. * *p* < 0.05, ** *p* < 0.01, *** *p* < 0.001, ns, not significant.

Post-antibiotic treatment, serum ALT, AST, and ALP levels remained significantly elevated in the ANTI group compared to antibiotic controls (ACON). Compared to the ANTI group, SLB administration selectively reduced ALT levels (*p* < 0.05), whereas AST and ALP showed no significant alterations (Figure 9A–C).

Humoral immunity assessment revealed that compared to the ANTI group, SLB intervention partially restored serum IgA levels. However, IgG and IgM levels in the ANTIS + SLB group remained comparable to those in the ANTI group (Figure 9D–F). These observations collectively suggest intestinal microbiota contribute to SLB-mediated restoration of hepatic and immune homeostasis.

### 3.9. Effect of Silibinin on Nrf2/Keap1 Pathway-Related Protein and mRNA Expression After Antibiotic Clearance

The Nrf2/Keap1 signaling pathway critically regulates cellular antioxidant responses through ARE activation. To evaluate its role in D-gal-induced oxidative stress, hepatic expression of Nrf2/Keap1 pathway components was assessed at both protein and mRNA levels.

**Figure 10:**
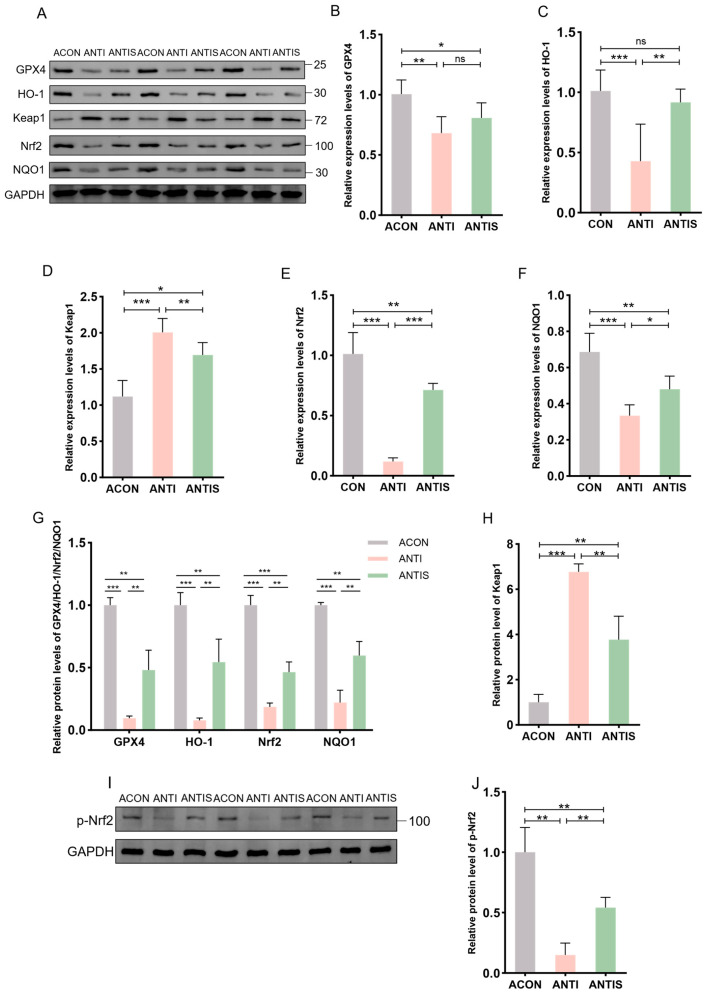
Nrf2 pathway responses in liver tissue of microbiota-depleted mice under D-galactose and silibinin interventions. (**A**,**I**) Western blot for GPX4, HO-1, Keap1, Nrf2, NQO1 and p-Nrf2 of murine liver tissue. (**B**–**F**) The mRNA abundance of Nrf2 signaling components (GPX4, HO-1, Keap1, NQO1) and Nrf2. (**G**,**H**,**J**) GPX4, HO-1, Nrf2, NQO1, Keap1 and protein expression was quantified. * *p* < 0.05, ** *p* < 0.01, *** *p* < 0.001, ns, not significant. GPX4 (glutathione peroxidase 4), HO-1 (nuclear heme oxygenase-1), NQO1 (NADH dehydrogenase quinone 1).

Compared to the ACON group, D-gal administration significantly suppressed Nrf2 protein expression while elevating Keap1 levels compared to controls (ACON). Consistently, phosphorylation of Nrf2 was similarly suppressed by D-gal challenge. In the ANTIS group, SLB treatment effectively restored nuclear Nrf2 translocation (by 2.5-fold) and p-Nrf2 expression, and reduced Keap1 accumulation (by 30%) compared to the ANTI group (Figure 10A,G–J). Downstream targets GPX4, HO-1, and NQO1 exhibited parallel protein suppression in the ANTI group relative to ACON levels. Although SLB significantly upregulated GPX4 expression (by 4.0-fold), HO-1 and NQO1 showed only partial restoration (4.0-fold and 2.0-fold increase, respectively) (Figure 10A,G).

At the mRNA level, relative to the ACON group, the ANTI group showed decreased expression of GPX4, HO-1, NQO1, and Nrf2 while increasing Keap1 expression. These transcriptional alterations were markedly normalized by SLB treatment in the ANTIS group compared to the ANTI group (Figure 10B–F).

### 3.10. Effects of Silibinin on the Composition of the Intestinal Flora as Well as Non-Target Metabolites in Mice with Oxidative Damage to D-Galactose After Antibiotic Removal

**Figure 11:**
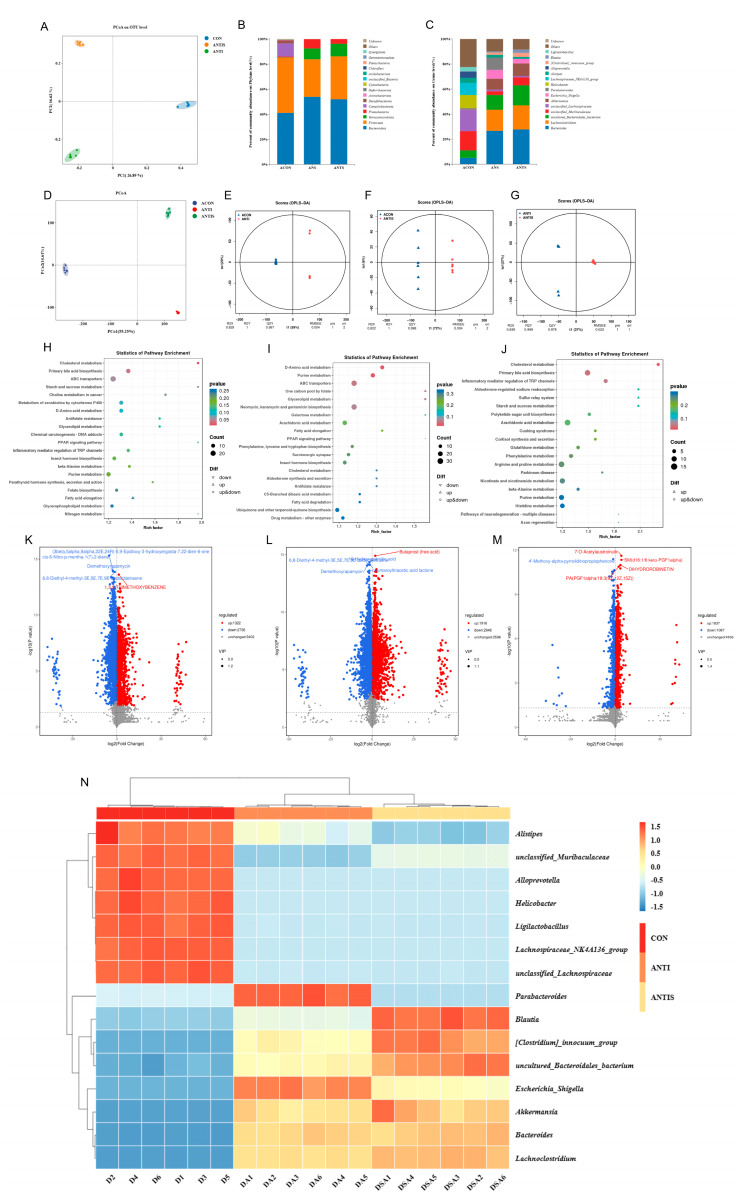
Multi-omics profiling of cecal microbiome and metabolome post-antibiotic microbiota depletion across control (ACON), antibiotic-treated (ANT), and antibiotic + silibinin (ANTS) groups. (**A**) Microbial β-diversity (PCoA). (**B**) Phylum-level relative abundance. (**C**) Genus-level relative abundance. (**D**) Metabolite β-diversity (PCoA) for pairwise comparisons. (**E**–**G**) OPLS-DA score plots. (**H**–**J**) KEGG enrichment of differential metabolites: ACON vs. ANT (**H**), ACON vs. ANTS (**I**), ANT vs. ANTS (**J**). (**K**–**M**) Volcano plots: ACON vs. ANT (**K**), ACON vs. ANTS (**L**), ANT vs. ANTS (**M**). (**N**) Heatmap of the relative abundance of gut microbiota at the genus level. Note that in panel (**L**), due to spatial constraints, the labels for two significant metabolites—6,8-Diethyl-4-methyl-3E,5E,7E,9E-dodecatetraene and 16-Hydroxypalmitic acid—are overlapping.

Alpha diversity analysis (Table 4) revealed significant reductions in Simpson, Shannon, ACE, and Chao1 indices in both ANTI and ANTIS groups compared to ACON (*p* < 0.05). ANTIS treatment partially restored community richness (increased ACE and Chao1 vs. ANTI) but further reduced diversity (decreased Simpson and Shannon vs. ANTI) (*p* < 0.05). Beta diversity analysis (PCoA, Figure 11A) confirmed significant structural differences among all groups (*p* < 0.05), indicating antibiotic-induced dysbiosis.

At the phylum level (Figure 11B, Table 5), ANTI group exhibited significantly increased Proteobacteria and Bacteroidota, but decreased Firmicutes versus ACON (*p* < 0.05). ANTIS group showed the highest Verrucomicrobiota abundance (*p* < 0.05 vs. ACON).

Genus-level analysis (Figure 11C, Table 5) demonstrated that ANTI group, relative to ACON, had increased *Bacteroides*, *Lachnoclostridiumbacterium*, *Akkermansia*, *Escherichia_Shigella*, *Parabacteroides*, *[Clostridium]_innocuum_group*, and *Blautia*, but decreased *unclassified_Muribaculaceae*, *unclassified_Lachnospiraceae*, *Helicobacter*, *Lachnospiraceae_NK4A136_group*, *Alistipes*, *Alloprevotella*, and *Ligilactobacillus* (*p* < 0.05). ANTIS intervention significantly modulated specific taxa versus ANTI group (*p* < 0.05). Notably, SLB intervention significantly boosted beneficial genera like *Lactobacillus* and *Akkermansia* compared to ANTI. These microbial shifts bolster gut barrier integrity and anti-inflammatory responses, primarily by *Akkermansia* strengthening mucosal protection and *Lactobacillus* contributing to SCFA production and pathogen inhibition [23,24].

Untargeted metabolomics detected 7460 metabolites, with pairwise comparisons identifying significant differential abundances: 4058 metabolites (1322 upregulated, 2736 downregulated) between ACON and ANTI groups; 4864 metabolites (1916 upregulated, 2948 downregulated) between ACON and ANTIS groups; and 2924 metabolites (1837 upregulated, 1087 downregulated) between ANTI and ANTIS groups. Multivariate analysis confirmed distinct metabolic segregation across groups via PCoA, while OPLS-DA validated significant separations in all comparisons (Figure 11E–G) with robust model parameters (R^2^Y ≥ 0.999, Q^2^Y ≥ 0.978). Volcano plots visualized these differential distributions (Figure 11K–M). KEGG enrichment analysis (*p* < 0.05) identified oxidative stress-associated pathways including Cholesterol metabolism, Primary bile acid biosynthesis, D-Amino acid metabolism, Purine metabolism, and Inflammatory mediator regulation of TRP channels (Figure 11H–J, Table 6). Crucially, ANTIS intervention significantly modulated key metabolites relative to ANTI group: Cholesterol metabolism exhibited altered Taurochenodeoxycholate (0.5 < |log_2_FC| < 1); Primary bile acid biosynthesis featured changes in Taurochenodeoxycholate, Choloyl-CoA, and 3α,7α,12α-Trihydroxy-5β-cholestanoate (|log_2_FC| > 1); and TRP channel regulation showed differential abundance of 5(S)-HETE, Icilin, Cinnamaldehyde, and 3′,5′-Cyclic AMP (0.5 < |log_2_FC| < 1) (all *p* < 0.05; Tables S4–S6). Notably, the observed modulation of bile acid metabolism (e.g., Taurochenodeoxycholate) and inflammatory mediators (e.g., 5(S)-HETE) by SLB intervention suggests a potential mechanism through which gut microbiota-dependent metabolic reprogramming may alleviate hepatic oxidative stress and restore liver function [25].

## 4. Discussion

Silibinin, as a natural flavonolignan, has been widely used in hepatoprotection and metabolic disorder-related disease intervention due to its remarkable antioxidant and anti-inflammatory properties. Studies have shown that it can alleviate oxidative damage through mechanisms such as scavenging free radicals, modulating the Nrf2/ARE signaling pathway and inhibiting lipid peroxidation. An important consideration for the translational relevance of our findings is whether the concentration of SLB used in this study is achievable in vivo. Pharmacokinetic studies have demonstrated that oral administration of silibinin at doses comparable to those used here can achieve plasma and tissue concentrations sufficient to elicit antioxidant and anti-inflammatory effects. For instance, one study revealed that orally administered silibinin reaches high plasma concentrations, characterized by a rapid distribution phase followed by a slower elimination phase, which enables effective antioxidant responses in various models [26]. Therefore, the dosage employed in our experiment is physiologically relevant and consistent with the efficacious levels reported in the literature.

In the systemic oxidative stress model induced by D-gal, the specific mechanism of SLB on multiorgan dysfunction, particularly its synergistic regulation of the liver–gut axis, remains unclear. Its potential value in slowing aging-related pathological processes also requires further exploration. In this experiment, to clarify the microbiota-dependent mechanism of SLB in alleviating D-gal-induced oxidative stress, a model of antibiotic depletion of intestinal microbiota was constructed. The study revealed that the efficacy of SLB significantly depended on metabolic interactions and signaling regulation mediated by the intestinal microbiota. Antibiotic treatment led to the depletion of butyrate-producing bacteria, such as *Muribaculaceae*, and secondary bile acid-converting bacteria, such as *Parabacteroides*. This disruption impaired the balance between short-chain fatty acid (SCFA) synthesis and bile acid metabolism, which in turn limited the activation of the Nrf2/Keap1 pathway and the restoration of antioxidant enzymes, including HO-1 and GPX4. Although SLB partially remodeled microbiota structure, for example, by enriching *Akkermansia* and *Bacteroides*, its intended microbiota functions, such as butyric acid production and tryptophan metabolism, were not compensated. Metabolomic analysis showed that accumulation of the primary bile acid Taurochenodeoxycholate was associated with upregulation of the mitochondrial toxic metabolite 3α,7α,12α-trihydroxy cholesterylate. Together, these changes exacerbated oxidative damage.

The hepatoprotective effect of SLB was confirmed through the synergistic operation of a “microbiota–metabolism–antioxidant” network.

D-gal, a reducing sugar naturally present in biological systems and dietary sources, is rapidly metabolized to glucose through the Leloir pathway and efficiently excreted within 8 h under physiological concentrations [27]. However, excessive exogenous D-gal intake induces pathological conversion to aldose and hydrogen peroxide via galactose oxidase activity. This process directly disrupts the oxidant-antioxidant equilibrium, initiating oxidative stress and hepatic dysfunction [11,28].Mechanistically, ROS generated during oxidative stress damage hepatocyte phospholipid bilayers, compromising membrane integrity [29,30]. Consequently, liver-specific enzymes ALT, AST, and ALP—are released into systemic circulation, elevating serum enzyme levels and propagating systemic oxidative stress [31,32]. Chronic oxidative stress further triggers a self-perpetuating cycle of antioxidant system failure. Sustained ROS production progressively inhibits critical antioxidant enzymes including SOD and CAT, while depleting reduced GSH reserves [31,33]. This cascade impairs free radical scavenging capacity and promotes lipid peroxidation. The resultant accumulation of MDA, a terminal lipid peroxidation product, induces mitochondrial dysfunction [34]. Histopathological manifestations include hepatocyte necrosis, vacuolar degeneration, inflammatory infiltration, and pseudolobule formation observable through H&E staining [35]. These structural alterations correlate with significant organ index reduction secondary to parenchymal loss [36].

Concurrently, oxidative stress exerts direct and indirect regulatory effects on immune system functionality. Immune organs, serving as primary sites for immune responses, execute critical immune defense mechanisms to effectively eliminate pathogens and detrimental stimuli. Notably, thymic and splenic indices represent key biomarkers for assessing immune competence. The thymus, functioning as a central immune organ, orchestrates the regulation of peripheral immune organs and immune cells while serving as the primary site for T cell differentiation and maturation. Moreover, the spleen, as a secondary lymphoid organ, not only provides the microenvironment for T and B cell residence and immune activation but also synthesizes and secretes diverse immunologically active mediators, fulfilling essential biological functions [37,38,39]. The pronounced decline in thymic and splenic indices among D-gal-exposed mice reflects systemic immune dysfunction induced by oxidative stress. This compromised immunity was paralleled by significant decreases in serum immunoglobulins (IgA, IgG, and IgM), with mechanistic underpinnings rooted in chronic D-gal exposure [40]. Chronic splenic impairment, mediated through B cell proliferation inhibition and apoptosis induction, directly disrupts IgM and IgG biosynthesis [41]. Concurrent intestinal oxidative damage and microbial dysbiosis further undermine IgA secretion by lamina propria B cells, while subsequent pathogen translocation perpetuates a self-reinforcing cycle of systemic inflammation and oxidative stress aggravation [42]. As the dominant bioactive compound in silymarin, SLB counteracts oxidative injury via dual mechanisms: direct scavenging of ROS/RNS coupled with suppression of lipid peroxidation biomarkers, thereby alleviating oxidative tissue damage [43].

We designed the D-gal + SLB group in our experiment to verify the mitigating effect of SLB on oxidative stress, and the results showed that SLB significantly reduced serum levels of transaminases and alkaline phosphatase and attenuated the level of lipid peroxidation increased the activity of related antioxidant enzymes and ameliorated the liver parenchymal lesions [44,45]. In addition, it has been shown that feed supplementation with SLB significantly increased serum IgA and IgM levels in broiler ducks, while the levels of pro-inflammatory factors TNF-α and IL-6 were significantly reduced, and this alteration of the inflammatory microenvironment may provide favorable conditions for the production of immunoglobulins and their immune function [46,47]. As a pivotal antibody in mucosal immunity, elevated IgA levels may strengthen host defense against pathogenic invasion. Our experiments demonstrated that SLB positively modulated serum immunoglobulin profiles, exhibiting dual immunoregulatory and anti-inflammatory properties that synergistically ameliorated systemic oxidative stress [48,49]. These findings collectively suggest that SLB’s hepatoprotective efficacy is mechanistically linked to its antioxidant capacity and immunomodulatory potential.

The enterohepatic axis mediates bidirectional communication between the gut and liver through dynamic metabolic exchange. Gut-derived nutrients, microbial metabolites (such as SCFAs), and endotoxins reach the liver via portal circulation. Against this physiological backdrop, we demonstrate that SLB activates the Nrf2/Keap1 pathway—a central cellular antioxidant regulator—through microbiota-dependent mechanisms. SLB-induced gut microbiota remodeling (marked by reduced *Firmicutes*/*Bacteroidetes* (F/B) ratio and *Lactobacillus* enrichment) initiates a dual defense strategy: (1) SCFA-producing commensals directly enhance antioxidant capacity, while (2) bacterial metabolites and SLB cooperatively activate Nrf2-driven cytoprotection.

Administration of SLB induced profound remodeling of the gut microbiota in D-gal-challenged mice, characterized by a dominance of *Firmicutes* and *Bacteroidetes*. This treatment significantly reduced the (F/B) ratio, which correlated with the attenuation of systemic oxidative stress [50]. The shift in microbial composition suppressed pro-oxidative bacterial taxa while enriching commensals that produce short-chain fatty acids (SCFAs) [50]. Increased levels of SCFAs, particularly butyrate and acetate, enhanced the activities of antioxidant enzymes such as SOD and GSH-Px, and suppressed ROS and MDA levels [51,52]. Mechanistically, the decreased F/B ratio alleviates redox imbalance through two main pathways: direct antioxidant activation via SCFAs and indirect suppression of pro-oxidant metabolites [53].

Furthermore, SLB treatment significantly activated the Nrf2/Keap1 pathway, a central regulatory system for cellular antioxidant responses [20]. Under oxidative stress, SLB promoted the dissociation of Nrf2 from Keap1, facilitating its nuclear translocation and binding to antioxidant response elements (AREs) [54]. This activation was further evidenced by a marked increase in Nrf2 phosphorylation, consistent with the upregulation of total Nrf2 protein and mRNA expression, along with induction of downstream effectors including NQO1, HO-1, and GPX4 [54]. Notably, enrichment of *Lactobacillus*—a genus known for its ability to scavenge reactive oxygen species through Nrf2 activation—amplifies this pathway via bacterial metabolite-mediated Nrf2 stimulation [55,56].

Taxonomic analysis identified *Lactobacillus* as the predominant genus enriched by SLB [57], indicating an ecological shift toward antioxidant-competent microbial strains. This establishes a bidirectional synergy: bacterial metabolites activate Nrf2 signaling, thereby augmenting the expression of SOD and CAT [56], while SLB directly targets Keap1, creating a cascading amplification effect. Through coordinated ROS neutralization and mitochondrial functional recovery, this dual mechanism provides comprehensive cytoprotection [58].

Metabolomic analyses revealed that SLB reprograms the cecal metabolome through targeted microbial and metabolic regulation. Elevated succinate levels indicated mitochondrial functional restoration, which is linked to Nrf2 activation via inhibition of Keap1 ubiquitination—promoting Nrf2 nuclear translocation and upregulating HO-1 and NQO1 [58,59,60]—as well as HIF-1α stabilization. Concurrent accumulation of 2-oxoglutaramate suggests nitrogen metabolic reprogramming [61], while increased 15(S)-HPETE correlates with NF-κB suppression [62,63]. A rise in 3-beta-D-galactosyl-sn-glycerol implies enhanced D-gal catabolism mediated by *Lactobacillus* proliferation [61,62,63]. Depletion of L-glutamine may reflect microbial adaptation or enhanced glutathione biosynthesis [64], and reduced icilin levels correspond to TRPM8-mediated anti-inflammatory effects [65,66]. Collectively, these metabolic shifts form an integrated defense network that encompasses antioxidant activation, resolution of inflammation, and enhanced detoxification of xenobiotics.

Immunoglobulin restoration involves complementary mechanisms—enhancement of B-cell functionality through Nrf2-mediated NF-κB inhibition, coupled with stimulation of intestinal IgA secretion by short-chain fatty acids originating from gut microbiota. Collectively, the hepatoprotective effects of SLB emerge from an interconnected antioxidant-metabolic-immunological network, wherein metabolites fulfill dual roles as both biomarkers and functional mediators. This systematic integration elucidates polypharmacological action through multi-target engagement of the compound.

To delineate the microbiota-dependent mechanisms underlying SLB’s antioxidant actions, we employed a D-gal/antibiotic perturbation model. Our findings indicate that compromised microbial functionality—particularly in SCFA production, bile acid metabolism, and immunomodulatory coordination—critically attenuates SLB’s efficacy. This perturbation reveals intricate host–microbe metabolic interdependencies governing oxidative stress resolution along the gut–liver axis.

To investigate gut microbiota dependency in SLB’s antioxidant effects, we employed a D-gal/antibiotic composite model. Antibiotic administration severely depleted butyrate-producing *unclassified_Muribaculaceae* (*Bacteroidota*) [67,68] and compromised SLB efficacy. Although SLB partially reversed microbial shifts (elevating *Bacteroides*, *Akkermansia*, and *Firmicutes*), it failed to restore core metabolic functions—particularly butyrate biogenesis. This impaired butyrate-dependent activation of the Nrf2/Keap1 pathway, preventing normalization of HO-1, NQO1, and GPX4 activities [69]. Critically, as butyrogenic populations were nearly eradicated, SCFA-mediated antioxidant pathways became inoperative, thereby abolishing SLB’s microbiota-dependent protection and exacerbating oxidative damage [24].

Concurrently, antibiotic-induced depletion of *Parabacteroides* reduced the synthesis of secondary bile acids such as lithocholic acid [70], leading to the accumulation of primary bile acids including cholic acid and taurochenodeoxycholate [24]. This exacerbated mitochondrial dysfunction and suppressed Nrf2 signaling via FXR hyperactivation [25,71]. Metabolomic profiling confirmed dysregulation: taurochenodeoxycholate and chenodeoxycholoyl-CoA were upregulated, while choloyl-CoA decreased—reflecting disrupted microbial bile acid transformation [72]. Pathologically elevated 3α,7α,12α-trihydroxy-5β-cholestanoate inhibited mitochondrial β-oxidation, elevating ROS generation while suppressing SOD/GPx activities [24].

Although the reduction in *Escherichia_Shigella* diminished endotoxin leakage [70], the antioxidant effects of SLB remained dependent on immunomodulatory taxa such as *Lachnospiraceae_NK4A136_group*. Combined antibiotic/D-gal interventions catastrophically disrupted these regulatory axes. *Lachnoclostridium* generated pro-oxidants via aromatic amino acid metabolism [73], while *Bifidobacterium* extinction impaired glutathione precursor synthesis. Diminished 5(S)-HETE further compromised inflammatory resolution through dysregulated COX/LOX pathways [62,63], contributing to inadequate serum immunoglobulin recovery.

Furthermore, functional coordination between *Bacteroides* and *Clostridium* was constrained [73], attenuating tryptophan metabolism and AhR-mediated barrier repair [74]. Although SLB partially alleviated injury via direct ROS scavenging and *Akkermansia*-associated mucosal regeneration [23,75], persistent dysregulation of microbiota–host interactions—particularly SCFA depletion and bile acid imbalance—obstructed systemic antioxidant defense along the gut–liver axis [76]. Supporting this observation, microbiota ablation attenuated silymarin’s biotransformation into active derivatives (e.g., isosilybin A) [77], impairing Nrf2 activation [78] and bile acid regulation.

Our findings collectively demonstrate that SLB’s antioxidant efficacy is contingent upon microbiota functional integrity, where impaired butyrate-dependent Nrf2 activation compromises enzymatic defense systems. Concurrent bile acid dysregulation exacerbates mitochondrial dysfunction and inflammatory signaling, while disrupted immunometabolic coordination attenuates barrier protection. Critically, the breakdown of host–microbe co-metabolism—evidenced by defective xenobiotic transformation—uncouples gut–liver antioxidant synergy. This mechanistic cascade underscores microbiota homeostasis as a prerequisite for SLB-mediated oxidative stress resolution.

Our study clarifies SLB’s microbiota-dependent antioxidant mechanisms, yet acknowledges inherent constraints. The D-gal murine model, while mechanistically informative, may imperfectly reflect human oxidative stress pathologies; clinical or organoid validation would strengthen translatability. Definitive causal attribution remains challenging with antibiotic-depletion models, necessitating corroboration via fecal microbiota transplantation or gnotobiotic systems. Crucially, *Muribaculaceae* (butyrogenesis) and *Parabacteroides* (bile acid metabolism) are indispensable for SLB efficacy, highlighting therapeutic potential for targeted probiotics or engineered SLB–microbiota co-therapies. Future priorities should develop gut-stable SLB derivatives, evaluate combinational regimens (e.g., SLB + A. muciniphila) in metabolic steatohepatitis models, and map SLB–microbiota co-metabolite pharmacokinetics to bridge translational gaps.

Collectively, our findings establish that SLB’s antioxidant efficacy is centrally orchestrated by gut microbiota-mediated metabolic functions. The drug’s ability to mitigate systemic oxidative stress depends critically on: (i) microbial butyrogenesis activating the Nrf2 pathway, (ii) commensal-dependent bile acid transformation preventing mitochondrial dysfunction, and (iii) immunomodulatory metabolite networks resolving inflammation. This work provides mechanistic evidence that host-microbiome metabolic crosstalk fundamentally dictates SLB’s therapeutic outcomes, underscoring the necessity of evaluating microbial contributions in pharmacological interventions targeting oxidative stress-related pathologies.

## 5. Conclusions

**Figure 12:**
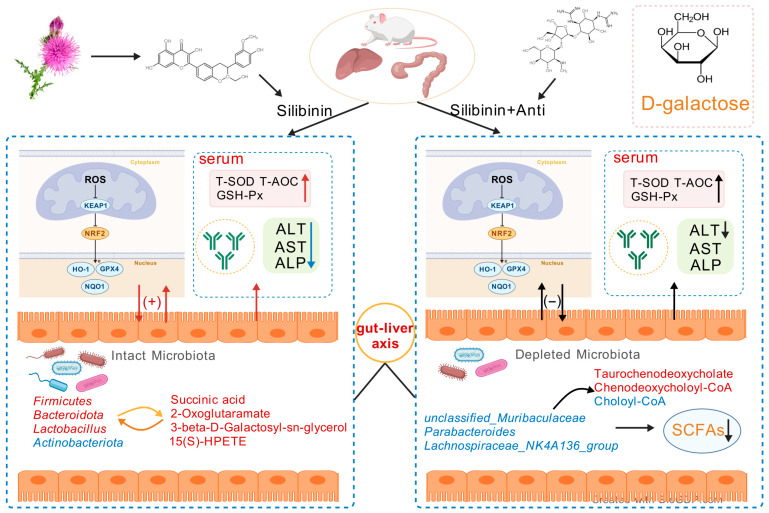
Schematic representation of the SLB–gut–liver–oxidative stress axis. The model depicts the potential of SLB to alleviate D-gal-induced hepatic oxidative stress in a microbiota-dependent manner by targeting the interplay between gut microbial ecology and host hepatic antioxidant pathways.

This study demonstrates that SLB protects against D-gal-induced hepatic oxidative stress in mice through multifaceted mechanisms: reducing ROS, enhancing antioxidant enzymes, restoring immunoglobulin profiles (IgA/IgM), and suppressing inflammatory cytokines. Critically, SLB’s efficacy depends on gut microbiota-mediated modulation of the gut–liver axis, as evidenced by antibiotic depletion experiments that abolished protection. These findings highlight SLB’s multidimensional actions, extending beyond antioxidative effects on immunomodulation. The mechanistic insights support developing microbiota-targeted therapies for age-related metabolic disorders—particularly those involving hepatic oxidative stress—where gut–liver dysregulation exacerbates redox imbalance (Figure 12). While precise molecular crosstalk warrants further investigation, targeting microbial–immune interactions represents a promising strategy for systemic antioxidant defense.

## Figures and Tables

**Table 1:** Effect of silibinin on microbial diversity index in cecal contents of mice with D-galactose-induced oxidative damage.

Item	Group			SEM	*p*-Value
	Control	D-Gal	SLB		
Shannon	1.90 ^b^	2.39 ^a^	2.45 ^a^	0.0533	<0.001
Simpson	0.31 ^a^	0.19 ^b^	0.17 ^b^	0.0160	<0.001
ace	282.64 ^c^	325.84 ^b^	356.12 ^a^	7.696	<0.001
chao1	283.40 ^c^	319.74 ^b^	354.21 ^a^	8.154	<0.001

Values with different superscript letters (a, b, c) denote significant differences (*p* < 0.05, *t*’s test).

**Table 2:** Abundance profiles of dominant cecal microbial taxa (phylum and genus levels) across experimental groups.

Item	Group			*p*-Value
	Control	D-Gal	SLB	
Phylum				
Firmicutes	95.21 ± 1.12 ^a^	86.44 ± 0.67 ^c^	91.58 ± 1.05 ^b^	<0.001
Actinobacteriota	3.50 ± 1.10 ^b^	10.78 ± 0.54 ^a^	2.43 ± 0.50 ^b^	<0.001
Bacteroidota	0.60 ± 0.27 ^c^	1.88 ± 0.38 ^b^	3.18 ± 0.98 ^a^	<0.001
Desulfobacterota	0.59 ± 0.073 ^c^	0.74 ± 0.083 ^b^	2.43 ± 0.39 ^a^	<0.001
Verrucomicrobiota	0.00 ± 0.00 ^b^	0.08 ± 0.04 ^b^	0.28 ± 0.15 ^a^	<0.001
Patescibacteria	0.08 ± 0.02 ^a^	0.04 ± 0.06 ^b^	0.05 ± 0.01 ^b^	0.002
Genus				
*Lactobacillus*	88.98 ± 2.64 ^a^	65.57 ± 1.29 ^c^	83.41 ± 3.70 ^b^	<0.001
*Bifidobacterium*	2.44 ± 1.10 ^b^	9.59 ± 0.50 ^a^	0.50 ± 0.21 ^c^	<0.001
*Turicibacter*	3.13 ± 1.31 ^b^	6.84 ± 0.60 ^a^	3.53 ± 1.94 ^b^	0.005
*Faecalibaculum*	0.79 ± 0.27 ^b^	9.65 ± 0.62 ^a^	0.19 ± 0.072 ^c^	<0.001
*norank_Muribaculaceae*	0.51 ± 0.24 ^c^	1.62 ± 0.33 ^b^	2.48 ± 0.86 ^a^	<0.001
*Desulfovibrio*	0.59 ± 0.070 ^c^	0.72 ± 0.085 ^b^	2.41 ± 0.39 ^a^	<0.001
*Enterorhabdus*	0.94 ± 0.079 ^c^	1.03 ± 0.082 ^b^	1.70 ± 0.30 ^a^	<0.001
*Romboutsia*	0.27 ± 0.12 ^b^	1.40 ± 0.084 ^a^	0.30 ± 0.13 ^b^	0.002
*unclassified_Lachnospiraceae*	0.58 ± 0.38 ^b^	0.60 ± 0.11 ^b^	1.18 ± 0.39 ^a^	0.02

Values with different superscript letters (a, b, c) denote significant differences (*p* < 0.05, *t*’s test).

**Table 3:** KEGG pathway enrichment analysis identified metabolites with significant differences among the CON, D-gal, and SLB groups.

ID	Description	Metabolite Ratio	Bg Ratio	Enrich Factor	*p*-Value
CON vs. D-Gal					
ko00650	Butanoate metabolism	2.0%	1.0%	1.95	0.01
ko00740	Riboflavin metabolism	2.0%	1.0%	1.95	0.01
ko00052	Galactose metabolism	1.0%	0.4%	2.44	0.03
CON vs. SLB					
ko00380	Tryptophan metabolism	5.4%	3.8%	1.41	0.02
ko05200	Pathways in cancer	2.6%	1.6%	1.60	0.03
ko04925	Aldosterone synthesis and secretion	1.2%	0.6%	1.94	0.05
D-gal vs. SLB					
ko00250	Alanine, aspartate and glutamate metabolism	2.1%	1.1%	1.87	0.01
ko04750	Inflammatory mediator regulation of TRP channels	2.3%	1.3%	1.76	0.02
ko00052	Galactose metabolism	0.9%	0.4%	2.28	0.04

**Table 4:** Silibinin effect on cecal microbial diversity in antibiotic-depleted, D-galactose-damaged mice.

Item	Group			SEM	*p*-Value
	ACON	ANTI	ANTIS		
Shannon	7.12 ^a^	4.87 ^b^	4.57 ^c^	0.0256	<0.001
Simpson	0.98 ^a^	0.94 ^b^	0.92 ^c^	0.0005	<0.001
ace	646.97 ^a^	191.93 ^c^	196.33 ^b^	35.0128	<0.001
chao1	642.00 ^a^	190.14 ^c^	194.03 ^b^	35.1574	<0.001

Values with different superscript letters (a, b, c) denote significant differences (*p* < 0.05, *t*’s test).

**Table 5:** Post-antibiotic abundance profiles of persistent cecal microbial taxa (phylum/genus) across intervention groups.

Item	Group			*p*-Value
	ACON	ANTI	ANTIS	
Phylum				
Bacteroidota	41.13 ± 0.46 ^c^	53.89 ± 0.97 ^a^	51.98 ± 1.81 ^b^	<0.001
Firmicutes	44.06 ± 0.62 ^a^	30.03 ± 0.76 ^c^	34.19 ± 0.69 ^b^	<0.001
Verrucomicrobiota	0.27 ± 0.04 ^c^	8.64 ± 0.64 ^b^	10.02 ± 1.27 ^a^	<0.001
Proteobacteria	0.62 ± 0.21 ^c^	7.34 ± 0.26 ^a^	3.78 ± 0.27 ^b^	<0.001
Campylobacterota	10.59 ± 0.45 ^a^	0.00 ± 0.00 ^b^	0.00 ± 0.00 ^b^	<0.001
Desulfobacterota	2.32 ± 0.14 ^a^	0.00 ± 0.00 ^b^	0.001 ± 0.002 ^b^	<0.001
Actinobacteriota	0.36 ± 0.11 ^a^	0.03 ± 0.02 ^b^	0.007 ± 0.007 ^b^	<0.001
Deferribacterota	0.23 ± 0.03 ^a^	0.00 ± 0.00 ^b^	0.00 ± 0.00 ^b^	<0.001
Cyanobacteria	0.20 ± 0.04 ^a^	0.002 ± 0.003 ^b^	0.00 ± 0.00 ^b^	<0.001
unclassified_Bacteria	0.04 ± 0.05 ^a^	0.02 ± 0.009 ^a^	0.006 ± 0.006 ^a^	0.19
Acidobacteriota	0.04 ± 0.04 ^a^	0.01 ± 0.01 ^ab^	0.00 ± 0.00 ^b^	0.08
Chloroflexi	0.03 ± 0.04 ^a^	0.006 ± 0.009 ^a^	0.003 ± 0.003 ^a^	0.19
Patescibacteria	0.03 ± 0.03 ^a^	0.002 ± 0.005 ^b^	0.001 ± 0.004 ^b^	0.02
Gemmatimonadota	0.02 ± 0.03 ^a^	0.002 ± 0.002 ^a^	0.002 ± 0.004 ^a^	0.29
Synergistota	0.01 ± 0.007 ^a^	0.005 ± 0.004 ^b^	0.00 ± 0.00 ^b^	0.003
Genus				
*Bacteroides*	5.00 ± 0.07 ^c^	26.63 ± 0.07 ^b^	27.74 ± 1.14 ^a^	<0.001
*Lachnoclostridiumbacterium*	0.08 ± 0.02 ^c^	16.82 ± 0.30 ^b^	19.23 ± 0.44 ^a^	<0.001
*uncultured_Bacteroidales_bacterium*	5.96 ± 0.35 ^c^	11.77 ± 0.34 ^b^	16.03 ± 0.65 ^a^	<0.001
*unclassified_Muribaculaceae*	15.28 ± 0.28 ^a^	3.01 ± 0.14 ^c^	6.26 ± 0.11 ^b^	<0.001
*unclassified_Lachnospiraceae*	17.13 ± 0.45 ^a^	1.39 ± 0.09 ^b^	1.22 ± 0.10 ^b^	<0.001
*Akkermansia*	0.26 ± 0.04 ^c^	8.64 ± 0.64 ^b^	10.02 ± 1.27 ^a^	<0.001
*Escherichia_Shigella*	0.05 ± 0.01 ^c^	7.08 ± 0.25 ^a^	3.77 ± 0.26 ^b^	<0.001
*Parabacteroides*	0.94 ± 0.07 ^b^	9.83 ± 0.25 ^a^	0.00 ± 0.00 ^c^	<0.001
*Helicobacter*	10.59 ± 0.46 ^a^	0.00 ± 0.00 ^b^	0.00 ± 0.00 ^b^	<0.001
*Lachnospiraceae_NK4 A136_group*	9.58 ± 0.25 ^a^	0.00 ± 0.00 ^b^	0.00 ± 0.00 ^b^	<0.001
*Alistipes*	4.03 ± 0.21 ^a^	2.20 ± 0.21 ^b^	1.43 ± 0.08 ^c^	<0.001
*Alloprevotella*	5.10 ± 0.24 ^a^	0.00 ± 0.00 ^b^	0.00 ± 0.00 ^b^	<0.001
*[Clostridium]_innocuum_group*	0.00 ± 0.00 ^c^	1.81 ± 0.15 ^b^	3.20 ± 0.23 ^a^	<0.001
*Blautia*	0.20 ± 0.03 ^c^	0.92 ± 0.07 ^b^	2.83 ± 0.10 ^a^	<0.001
*Ligilactobacillus*	3.35 ± 0.10 ^a^	0.02 ± 0.02 ^b^	0.004 ± 0.01 ^b^	<0.001

Values with different superscript letters (a, b, c) denote significant differences (*p* < 0.05, *t*’s test).

**Table 6:** KEGG pathway enrichment analysis identified metabolites with significant differences among the ACON, ANTI, and ANTIS groups.

ID	Description	Metabolite Ratio	Bg Ratio	Enrich Factor	*p*-Value
ACON vs. ANTI					
ko04979	Cholesterol metabolism	1.2%	0.6%	1.97	0.02
ko00120	Primary bile acid biosynthesis	3.6%	2.6%	1.37	0.04
ACON vs. ANTIS					
ko00470	D-Amino acid metabolism	2.8%	2.1%	1.33	0.03
ko00230	Purine metabolism	3.6%	2.8%	1.28	0.03
ANTI vs. ANTIS					
ko04979	Cholesterol metabolism	1.4%	0.6%	2.31	0.03
ko00120	Primary bile acid biosynthesis	3.9%	2.6%	1.49	0.05
ko04750	Inflammatory mediator regulation of TRP channels	2.2%	1.3%	1.70	0.05

## Data Availability

The authors confirm that the data supporting the findings of this study are available at PRJNA1283564 and PRJNA1283580 (NCBI).

## Supplementary Materials

The following supporting information can be downloaded at: https://www.mdpi.com/article/10.3390/antiox14091087/s1, Table S1: Identification of significantly different metabolites between CON group and D-gal group. Table S2: Identification of significantly different metabolites between CON group and SLB group. Table S3: Identification of significantly different metabolites between D-gal group and SLB group. Table S4: Identification of significantly different metabolites between ACON group and ANTI group. Table S5: Identification of significantly different metabolites between ACON group and ANTIS group. Table S6: Identification of significantly different metabolites between ANTI group and ANTIS group. Table S7: Sequences of primers used for RT-PCR.
