# Supplementary material for: Silibinin Alleviates Liver Oxidative Stress in D-Galactose-Treated Kunming Mice via Microbiota Homeostasis in a Microbiota-Dependent Manner
Source: Antioxidants (Basel). 2025 Sep 5;14(9):1087. doi: 10.3390/antiox14091087 (PMC12466601; doi:10.3390/antiox14091087)
Supplement: Supplementary file 1 [file antioxidants-14-01087-s001.zip › antioxidants-3798828-supplementary.pdf]

Table S1. Identification of significantly different metabolites between CON group and D-gal group.

KEGG pathway ID	metabolite ID	metabolite name	FC	*P*-value	VIP	CON vs. D-gal
ko00650	neg_1525	(R)-3-Hydroxybutanoate	1.2855	0.004	1.2582	up
	neg_2072	Fumarate	1.3855	0.016	1.0848	up
	neg_4864	Oxoglutaric acid	0.6428	0.001	1.3699	down
	neg_5591	Butanoic acid	<0.001	0.012	1.2206	down
	neg_7991	2-Oxoglutarate	1.3122	0.008	1.1492	up
	neg_960	Maleic acid	0.8885	0.023	1.0102	down
	pos_8612	3-Butyn-1-al	1.1192	<0.001	1.4278	up
	pos_991	Diacetyl	1.2797	<0.001	1.4873	up
ko00740	neg_1856	5-Amino-6-(5'-phosphoribosylamino)uracil	2.6285	<0.001	1.4595	up
	neg_4456	FAD	1.1333	0.006	1.2405	up
	neg_8858	6,7-Dimethyl-8-(D-ribityl)lumazine	0.1038	<0.001	1.4671	down
	neg_9278	Reduced FMN	23.8706	0.013	1.2148	up
	pos_11108	Prenol	0.6821	<0.001	1.4986	down
	pos_4104	FMN	0.6927	0.003	1.2250	down
	pos_7545	7-Hydroxy-6-methyl-8-ribityllumazine	3.0246	<0.001	1.4651	up
	pos_7919	2,5-Diamino-6-(5-phospho-D-ribosylamino)pyrimidin-4(3H)-one	1.5529	<0.001	1.5461	up
ko00052	neg_7616	Melibiitol	1.1507	<0.001	1.3992	up
	neg_806	Stachyose	0.6277	<0.001	1.5413	down
	neg_9755	Raffinose	1.1605	0.027	1.0462	up
	pos_4310	3-beta-D-Galactosyl-sn-glycerol	0.6289	<0.001	1.3710	down

Table S2. Identification of significantly different metabolites between CON group and SLB group.

KEGG pathway ID	metabolite ID	metabolite name	FC	*P*-value	VIP	CON vs. SLB
ko00380	neg_12779	2-Aminomuconate semialdehyde	0.0470	0.004	1.2308	down
	neg_194	Glucobrassicin	0.6805	0.002	1.2928	up
	neg_4147	Xanthurenic acid	1.5315	<0.001	1.3953	down
	neg_4445	Indolepyruvate	2.2125	<0.001	1.4195	up
	neg_5078	Indolylmethyl-desulfoglucosinolate	2.1635	<0.001	1.2813	up
	neg_5182	Formyl-5-hydroxykynurenamine	2.0464	0.014	1.0739	up
	neg_5381	Skatole	0.6784	0.001	1.2624	down
	neg_928	5-(3'-Carboxy-3'-oxopropyl)-4,6-dihydroxypicolinate	1.4628	<0.001	1.3933	up
	pos_11169	3-Indoleacetonitrile	1.4330	<0.001	1.3886	up
	pos_11414	5-Methoxytryptamine	1.6685	0.014	1.0335	up
	pos_40	N-Methylserotonin	0.5350	<0.001	1.4348	down
ko05200	neg_12497	Prostaglandin E2	0.6821	<0.001	1.4439	up

	neg_2799	Retinoate	0.6927	0.027	1.0158	down
	pos_1630	Dihydrotestosterone	3.0246	<0.001	1.3900	down
	pos_9075	4-Hydroxynonenal	1.5529	<0.001	1.4205	up
	pos_9322	Cortisone	1.1507	<0.001	1.3921	up
ko04925	neg_14519	Pregnenolone	1.5230	0.006	1.1640	up
	neg_5877	corticosterone	1.2133	0.003	1.1750	up
	pos_1988	Cholesterol	1.3069	<0.001	1.4170	up
	pos_5036	Aldosterone	0.8757	<0.001	1.3118	down
	pos_9166	Arachidonate	1.1539	0.001	1.2577	up

Table S3. Identification of significantly different metabolites between D-gal group and SLB group.

KEGG pathway ID	metabolite ID	metabolite name	FC	*P*-value	VIP	D-gal vs. SLB
ko00250	neg_1314	Succinic acid	1.4636	<0.001	1.2740	up
	pos_6970	2-Oxoglutaramate	7.6285	<0.001	1.5189	up
	pos_765	L-Glutamine	0.4875	<0.001	1.4986	down
ko04750	neg_16592	15(S)-HPETE	2.1836	0.03	1.0493	up
	pos_11998	Icilin	0.4168	<0.001	1.4564	down
ko00052	neg_9755	Raffinose	0.7510	0.002	1.2143	down
	pos_4310	3-beta-D-Galactosyl-sn-glycerol	1.6188	<0.001	1.3545	up

Table S4. Identification of significantly different metabolites between ACON group and ANTI group.

KEGG pathway ID	metabolite ID	metabolite name	FC	*P*-value	VIP	ACON vs ANTI
ko04979	pos_13698	Glycochenodeoxycholate	1.3021	<0.001	1.2534	up
	pos_1988	Cholesterol	0.5137	<0.001	1.2835	down
	pos_6420	Taurocholate	0.7579	<0.001	1.2873	down
	pos_7287	Glycocholate	0.6772	<0.001	1.2819	down
	pos_7373	Taurochenodeoxycholate	0.2333	<0.001	1.2953	down
	pos_7468	Taurocholic acid	1.2891	<0.001	1.2675	up
ko00120	neg_1189	Chenodeoxycholoyl-CoA	0.3330	<0.001	1.0985	down
	neg_13691	3alpha,7alpha,12alpha,26-Tetrahydroxy-5beta-cholestane	0.2003	<0.001	1.1076	down
	neg_14851	7alpha,12alpha-Dihydroxy-5beta-cholestan-3-one	0.6016	<0.001	1.0883	down
	neg_16421	3beta,7alpha-Dihydroxy-5-cholestenoate	3.9273	0.006	1.0972	up
	pos_10474	7alpha-Hydroxy-3-oxo-4-cholestenoate	0.4651	<0.001	1.2932	down
	pos_12427	3alpha,7alpha-Dihydroxy-5beta-cholestan-26-al	0.6528	0.013	1.2952	down
	pos_3773	(25R)-3alpha,7alpha-Dihydroxy-5beta-cholestanoyl-CoA	2.3229	<0.001	1.2352	up
	pos_6420	Taurocholate	0.7579	0.003	1.2873	down
	pos_7006	7alpha,27-Dihydroxycholesterol	1.6487	<0.001	1.2090	up
	pos_7287	Glycocholate	0.6772	<0.001	1.2819	down
	pos_7373	Taurochenodeoxycholate	0.2333	<0.001	1.2953	down
	pos_9840	3alpha,7alpha,12alpha-Trihydroxy-5beta-cholestanoate	0.4141	<0.001	1.2845	down
	pos_9166	Arachidonate	1.1539	0.001	1.2577	up

Table S5. Identification of significantly different metabolites between ACON group and ANTIS group.

KEGG pathway ID	metabolite ID	metabolite name	FC	*P*-value	VIP	ACON vs. ANTIS
ko00470	neg_1078	Trans-4-Hydroxy-L-proline	0.5699	<0.001	1.1189	down
	neg_1982	N-Acetyl-D-phenylalanine	0.2924	<0.001	1.1800	down
	neg_6631	2-Oxoarginine	0.0133	<0.001	1.0920	down
	neg_6691	L-Phenylalanine	0.0437	<0.001	1.1673	down
	neg_706	Hydroxyproline	0.6970	<0.001	1.1239	down
	neg_7991	2-Oxoglutarate	0.2201	<0.001	1.1269	down
	pos_10849	L-Serine	3.3370	<0.001	1.1724	up
	pos_4239	(2R,4S)-2,4-Diaminopentanoate	0.1275	<0.001	1.1648	down
	pos_4366	L-Methionine	985.31	<0.001	1.1747	up
	pos_4910	L-Ornithine	0.6796	<0.001	1.0639	down
	pos_5102	L-Histidine	30.832	<0.001	1.1734	up
	pos_540	D-Proline	0.6285	<0.001	1.1551	down
	pos_704	L-Proline	0.5319	<0.001	1.1657	down
	pos_7219	D-Lombricine	5.2991	<0.001	1.1753	up
	pos_765	L-Glutamine	2.6376	<0.001	1.0999	up
	pos_8720	5-Aminopentanoate	0.0081	<0.001	1.1309	down
ko00230	neg_1079	Hypoxanthine	0.2908	<0.001	1.1735	down
	neg_386	Sulfate	2.2064	<0.001	1.1618	up
	neg_4295	Xanthosine 5'-phosphate	39906	0.002	1.0529	up
	neg_5072	Allantoic acid	16.713	<0.001	1.1734	up
	neg_5126	5-Aminoimidazole	47876	<0.001	1.1803	up
	neg_5204	Urate	11999	<0.001	1.1816	up
	neg_6129	1-(5-Phospho-D-ribosyl)-5-amino-4-imidazolecarboxylate	303.31	<0.001	1.1764	up
	neg_6343	1-(5'-Phosphoribosyl)-5-formamido-4-imidazolecarboxamide	686.34	<0.001	1.1440	up
	neg_6622	5'-Butyrylphosphoinosine	11.159	<0.001	1.1056	up
	neg_7050	dGMP	37.648	<0.001	1.1659	up
	pos_10108	Aminoimidazole ribotide	92634	<0.001	1.1685	up
	pos_4693	Deoxyinosine	1.7173	<0.001	1.0829	up
	pos_478	5-Hydroxy-2-oxo-4-ureido-2,5-dihydro-1H-imidazole-5-carboxylate	0.1058	<0.001	1.1487	down
	pos_509	2'-Deoxyguanosine 5'-monophosphate	0.4946	<0.001	1.1774	down
	pos_5594	1-(5'-Phosphoribosyl)-5-amino-4-imidazolecarboxamide	4.3651	<0.001	1.1177	up
	pos_573	5'-Phosphoribosylglycinamide	4.8164	<0.001	1.1737	up
	pos_672	3',5'-Cyclic AMP	0.0679	<0.001	1.1594	down
	pos_765	L-Glutamine	2.6376	<0.001	1.0999	up
	pos_889	5'-Phosphoribosyl-N-formylglycinamide	5.6551	<0.001	1.1789	up

Table S6. Identification of significantly different metabolites between ANTI group and ANTIS group.

KEGG pathway ID	metabolite ID	metabolite name	FC	*P*-value	VIP	ANTI vs. ANTIS
ko00470	pos_13698	Glycochenodeoxycholate	0.8855	<0.001	1.4112	down
	pos_1988	Cholesterol	0.7331	<0.001	1.4382	down
	pos_6420	Taurocholate	1.2831	<0.001	1.5520	up
	pos_7287	Glycocholate	1.1487	<0.001	1.3896	up
	pos_7373	Taurochenodeoxycholate	2.3410	<0.001	1.6259	up
ko00230	neg_1189	Chenodeoxycholoyl-CoA	1.8751	0.04	1.0667	up
	neg_16681	3alpha,7alpha-Dihydroxy-5beta-cholestanate	1.4246	0.04	1.0836	up
	pos_7006	7alpha,27-Dihydroxycholesterol	0.5602	<0.001	1.4258	down
	pos_7373	Taurochenodeoxycholate	2.3410	<0.001	1.6259	up
	pos_9632	Choloyl-CoA	0.4089	0.005	1.2654	down
	pos_9840	3alpha,7alpha,12alpha-Trihydroxy-5beta-cholestanoate	2.0630	<0.001	1.6066	up
ko04750	neg_9219	5(S)-HETE	0.4537	0.02	1.1892	down
	pos_11998	Icilin	0.5093	<0.001	1.4874	down
	pos_12631	(-)-Menthol	1.4442	<0.001	1.5040	up
	pos_5214	Cinnamaldehyde	9.3145	0.003	1.3602	up
	pos_672	3',5'-Cyclic AMP	0.3610	0.01	1.1641	down

Table S7. Sequences of primers used for RT-PCR.

**Primer**	**Sequence**
β-actin F	TATGCTCTCCCTCACGCCATCC
β-actin R	GTCACGCACGATTTCCCTCTCAG
GPX-4 F	ATAAGAACGGCTGCGTGGTGAAG
GPX-4 R	TAGAGATAGCACGGCAGGTCCTTC
HO-1 F	ACCGCCTTCCTGCTCAACATTG
HO-1 R	CTCTGACGAAGTGACGCCATCTG
NQO1 F	GCGAGAAGAGCCCTGATTGTACTG
NQO1 R	AGCCTCTACAGCAGCCTCCTTC
Keap1 F	TGGTCGCCCTGTGCCTCTATG
Keap2 R	TCGTCCCGCTCTGGCTCATATC
Nrf2 F	TTGCCACCGCCAGGACTACAG
Nrf2 R	ACTTGTACCGCCTCGTCTGGAC
